# High sensitivity NH_3_ gas sensor with electrical readout made on paper with perovskite halide as sensor material

**DOI:** 10.1038/s41598-019-43961-6

**Published:** 2019-05-23

**Authors:** Avisek Maity, A. K. Raychaudhuri, Barnali Ghosh

**Affiliations:** 1 Department of Condensed Matter Physics and Materials Sciences, S.N. Bose National Centre for Basic Sciences, JD Block, Sec-III, Salt Lake, Kolkata 700106 India; 2 Technical Research Centre (TRC), S.N. Bose National Centre for Basic Sciences, JD Block, Sec-III, Salt Lake, Kolkata 700106 India

**Keywords:** Materials science, Sensors and biosensors

## Abstract

In this paper we report a cheap, paper electronics based solid state gas sensor to detect NH_3_ gas selectively with a detection capability of better than 1 ppm. The sensor uses perovskite halide CH_3_NH_3_PbI_3_ (MAPI) as the active sensor material grown on a paper. This paper based sensor works at room temperature. The current through the paper sensor increases by one order on exposure to only 10 ppm NH_3_ gas. The calibrated sensitivity is ~55% for 1 ppm of NH_3_ gas in Nitrogen or Air. The current noise limited resolution estimated to be ~10 ppb. This work establishes perovskite halide as a new solid state gas sensing material that can reach sub ppm sensitivity using simple paper electronics. Use of paper and also solution method used to grow the active material makes the sensor cost effective and easy to manufacture. This type of disposable high sensitive paper sensor can be used for detection of NH_3_ as a marker in exhaled breathes for non-invasive diagnosis. The sensor formed on the paper, since it supports unheated operation, needs less than few nanowatt power for its operation.

## Introduction

Thin film gas sensor for detection of toxic and hazardous gases is a well researched area and is growing rapidly due to application potentials and also for development of new materials, in particular, nanomaterials that have enhanced functionality. In recent past, several materials with their different nanostructures are being widely used for detection of harmful pollutants^1,2^. However, detection limit, working temperature, cost effective fabrication technique, calibration transferability etc are the major factors that often limit the usage of these gas sensors in real field applications. A new application potential for very high sensitivity gas sensor is in breathe analysis for early disease detection using exhaled breathe. Very high sensitive sensors for specific gases are needed for analysis of exhaled breathe that can detect markers for ailments in stomach, lung or in other body parts^3^. In this context paper electronics based sensors have a niche because of its cost-effectiveness, disposability and also wearability.

Paper based point-of-care (POC) devices are rapidly evolving for analytical and clinical applications^4^. There are considerable current interests on state-of-art health monitoring and early diagnosis of different diseases via analysis of biomarker in exhaled breathe. Several studies have been  reported on strong correlation between exhaled breathe and specific diseases^5^. For example exhaled NO gas is well studied as marker for oxidative stress, while exhaled CO is used as marker for diabetes, nephritis and bilirubin production^6,7^. Kidney failure which is a related to irreversible loss of kidney function is clinically silent up to a very advanced stage. However, pathology of this disease at early stage can be characterized by increment of ammonia (NH_3_) concentration of few hundreds of ppb (~100 ppb) level in exhaled breathe. NH_3_ gas has also been recognized as one of the marker for hepatic or kidney diseases^8^. Thus a disposable, cheap and easy to use paper based breath sensor with capability of NH_3_ detection well below 1 ppm is highly desirable for use as a diagnosis tool in breathe analysis.

In this paper, we report a paper based disposable sensor with a very high sensitivity that is able to detect NH_3_ gas of very low concentration well below ~1 ppm. The sensor is based on electrical read out and is workable at room temperature without need of any heating. The current noise limited detectability of the sensor is ~±10 ppb. This makes the sensor a viable tool not only for hazards environmental gas detector but also a tool for detection of NH_3_ as a disease marker in exhaled breathe. The innovation involves effective utilization of new materials like perovskite halide which has not been utilized effectively before for gas sensing.

Several reports are available on electrical read out based NH_3_ gas sensor using polyaniline (PANI), reduced graphene oxide (RGO)-ZnO, ZnO-SnO_2_^9–11^. However, to the best of our knowledge, till date no report is available on electrical read out NH_3_ sensor based on perovskite halide MAPI. The reported sensor works at room temperature and shows much higher sensitivity compared to those sensors reported before. A color change based NH_3_ gas sensor made using MAPI has been reported by our group before^12^ whereas, the present invention is based on electrical read out to trace the NH_3_ gas using same material.

There are serious challenges in detection of gases in exhaled human breathe due to suitable material and technologies that could selectively trace the specific markers and also allow disposability for use in individual testing. The detection capability needed is often 1 ppm or better. NH_3_ gas sensors are based on metal -oxide semiconductors (MOS) and PANI as active materials are generally used as environmental sensor. A few reports are available on breathe analyzer for NH_3_ detection in exhaled human breathe. They are based on MOS and needs elevated working temperature (200°–500 °C) for operation, have poor selectivity and show slow response- recovery rate^13^. It is thus important to investigate new classes of materials with very high sensitivity and selectivity that could be employed for exhaled breath analysis and also investigate whether they can be made compatible with cheap and disposable paper electronics with unheated operation capability. The present investigation demonstrates use of the perovskite halide, methyl ammonium lead iodide (CH_3_NH_3_PbI_3_ /MAPI) as a new material for sensing hazardous NH_3_ gas. The material for gas sensing  is grown on a paper and it can be used as unheated sensor. Being based on paper electronics, the sensor is disposable and can reach sub ppm level NH_3_ detection capability. Since, the paper sensor is being operated at 1 V DC with an output current in the range of only few nA, it requires very less (~nanowatt) power for operation. This makes it a low power consumption sensor, in contrast to commonly metal oxide sensors with typically milliwatt power consumption.

Hybrid halide perovskite especially methyl ammonium lead iodide (CH_3_NH_3_PbI_3_) or MAPI is a widely used material for photovoltaic^14^. But this material has not been much investigated as an active material for gas sensors. Till date report on sensing of ozone gas by mixed hybride halide perovskite is available^15^. Novelty of the present work is that it uses perovskite halide material for high sensitivity NH_3_ gas detection at room temperature and the material is compatible for use in paper electronics.

## Results

### Characterizations of the MAPI film on paper

Formation of MAPI film on paper has been reported by our group before^12^. The films were characterized by X-ray Diffraction (XRD), Field Emission Scanning Electron Microscope (FESEM) and Energy Dispersive Analysis of X-ray (EDX). The details are given in earlier publication and are not mentioned here to avoid duplication. Some of the relevant informations are given in Supplementary Materials.

**Figure 1:**
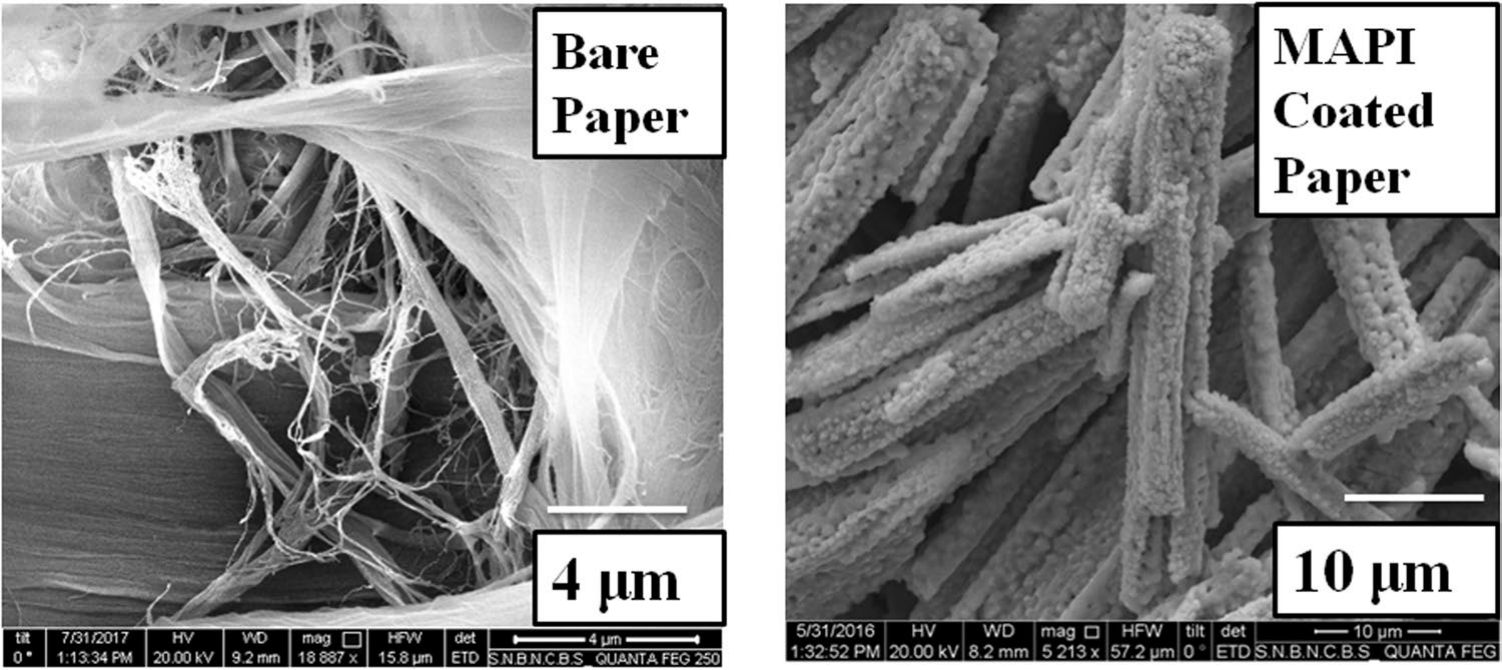
SEM image of the film with rod morphology along with the bare paper on which the material was grown and the sensor fabricated.

The important information for the present report refers to the morphology of the film. The film consists of nano and microrods of MAPI that grow on the paper substrate. FESEM image of the as grown MAPI film on the paper is shown in Fig. 1 along with the image of the bare paper.The bare paper has fibrous like structures arising from cellulose fibres. These fibres act as templates on which the MAPI film grows with nanorod morphology. The rods have diameter ranging from ~0.7 µm to 1.6 µm and average length is around 30 µm. The film is a dense array of the MAPI nano/micro rods.

### Electrical characteristics of MAPI film on paper

**Figure 2:**
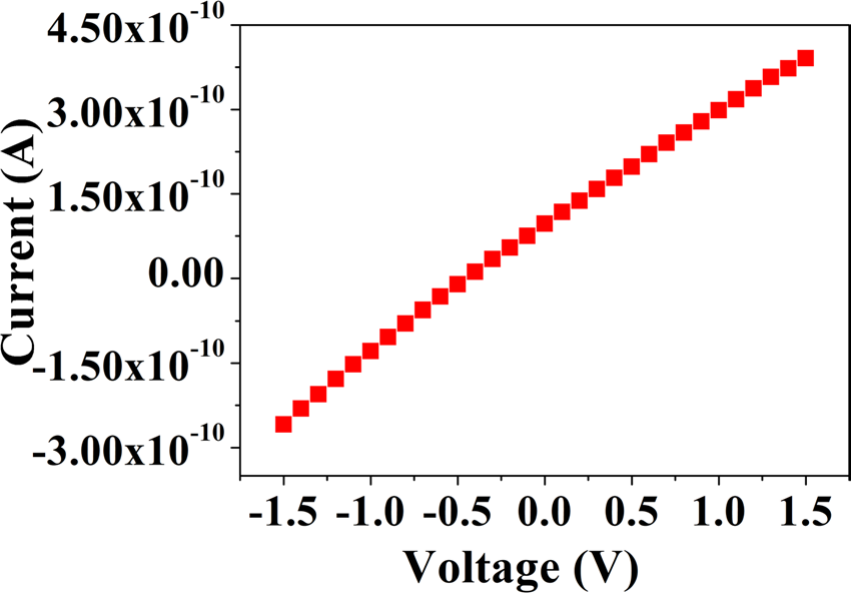
Linear I-V characteristics of the Sensor film using Cu electrodes.

**Figure 3:**
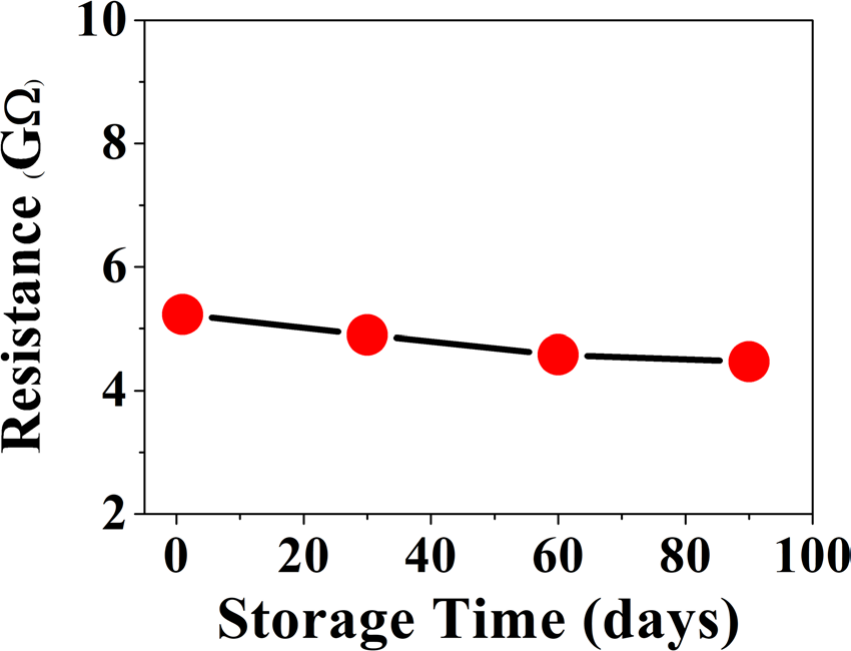
Stability of the MAPI film on paper substrate tested over 100 days period.

The electrical characteristic (I–V) of grown MAPI film is shown in Fig. 2. The linear I–V curve shown in Fig. 2 indicates ohmic nature of the Cu contact. A number of devices were tested to check the sensitivity of the paper sensor. It is crucial for any electrical sensor to check the stability of the base resistance of the sensors. The typical base resistance of the sensor varies from 4.5 GΩ to 7.5 GΩ depending on the details of the method of fabrication and sensor size. In general, average base resistance of the sensors is found to be 5.5 ± 1.1 GΩ. We have also tested the stability of the bare MAPI films towards storage (in a desiccator). We found that resistance of the films varies typically within ~±0.5 GΩ (10% of the average device resistance) over a storage period of 100 days. The data for the stability of the paper sensor are given in Fig. 3.

### Study of NH_3_ gas sensing property

(a) Sensitivity study of the sensor for exposure to low concentration of NH_3_ (≤10 ppm):

The devices were tested for exposure to NH_3_ gas with concentration ≤10 ppm (in dry N_2_ base gas). (We have also give data later on for test done for higher concentration NH_3_ gas for completeness).The flexible MAPI sensor was placed into the test chamber for investigation of its sensitivity towards NH_3_ gas.

**Figure 4:**
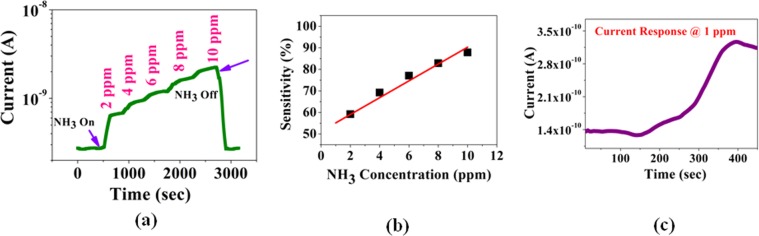
(**a**) Current response of the sensor for low NH_3_ concentration (2–10 ppm): Data taken with an applied bias of 1 V. (**b**) Response curve (S) as a function of gas concentration in range of 2 ppm to 10 ppm (**c**) Current response for 1 ppm NH_3_ gas concentration.

The initial test condition was set up by repeated evacuation and purging the test chamber with flowing dry nitrogen. A base atmosphere of dry nitrogen with known moles is created in the tested chamber. A calibrated amount (moles) of test gas (NH_3_) was then admitted. The test gas was admitted by steps of 2 ppm. (Data for 1 ppm exposure is given in Fig. 4(c)). The data on a representative device are shown in Fig. 4(a). After a concentration of 10 ppm was reached the chamber has been pumped out and dry N_2_ was admitted. This leads to recovery of the starting condition and the current through the device prior to the gas exposure is restored. It has been observed that with only 10 ppm NH_3_ gas the device current changes by nearly one order of magnitude.

The response (also often termed as sensitivity) of the sensor towards exposure to gas is defined as:

1 $$S\equiv \frac{({R}_{0}-{R}_{g})}{{R}_{0}}=\frac{{\rm{\Delta }}R}{{R}_{0}}$$

where *R*_*g*_ and *R*_0_ are the resistances of the sensor when exposed to NH_3_ gas and without gas (base resistance) respectively. In our case, the resistance of the device on gas exposure *R*_*g*_ < *R*_0_. The definition as in Eq. 1 gives a positive *S*. (Note: In certain cases where the gas exposure leads to *R*_*g*_ > *R*_0_, the order of *R*_*g*_ and *R*_0_ may be reversed to retain a positive *S*.)

Figure 4(b) shows the response $S$ of the MAPI sensor at different concentration of NH_3_ gas at room temperature from 1 ppm to 10 ppm. The dependence of $S$ on the NH_3_ in this range is linear. The test was carried out at room temperature. **(**b**)** Sensitivity of the Sensor for Exposure to Higher Concentration of NH_3_ (5 ppm–50 ppm).

**Figure 5:**
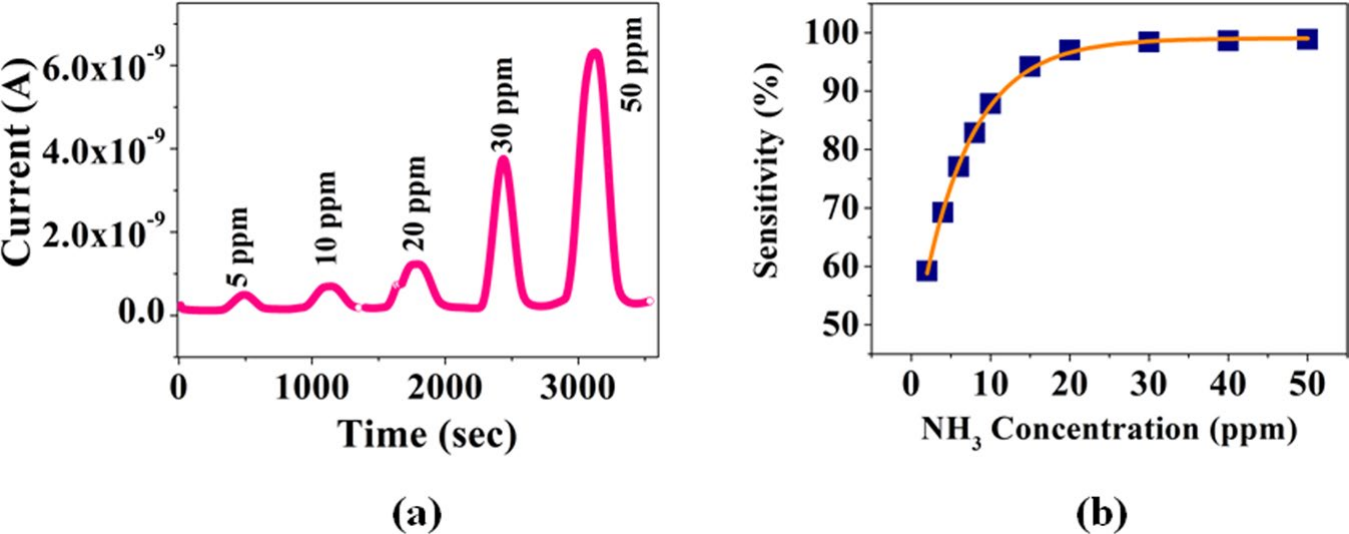
(**a**) Current response of the sensor for low ammonia concentration upto 50 ppm (**b**) Sensitivity as a function of NH_3_ gas concentration.

Although our focus is to study the low concentration regime of NH_3_ gas, but for a complete study of the response and recovery behaviour of the sensor, we recorded the response of the sensor exposed to different concentration of NH_3_ gas ranging from 5 ppm to high 50 ppm by repeating the gas ON-OFF cycle. At ON part we admitted the calibrated amount of gas in test chamber filled with dry N_2_. In the OFF cycle, we pumped out the chamber and admit dry nitrogen and get it ready for the next ON cycle. The device current response data and the sensitivity *S* are shown in Fig. 5(a) and 5(b) respectively. It can be seen that the response saturates beyond 30 ppm. It is also noteworthy that the previous report of visual sensing of NH_3_ gas by us was based on color change (detection limit ~10 ppm) whereas, in present case electrical sensor has much faster response (detection limit <1 ppm).

### Reproducibility and stability of the response

**Figure 6:**
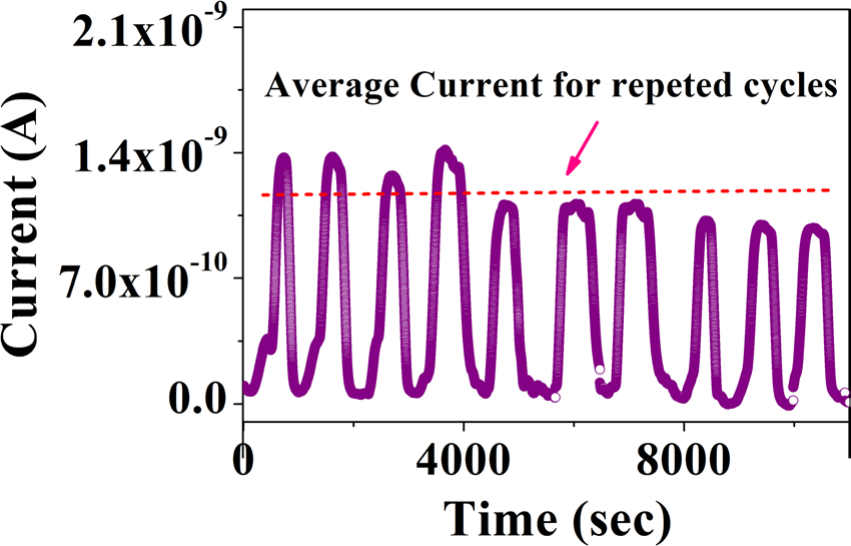
Repeatability of a particular sensor for 10 cycles for a fixed concentration of 10 ppm NH_3_ gas.

Experiments have also been performed for several cycles to study the repeatability / reproducibility of the sensor. It is observed that even after repeated cycles of exposure and recovery, for a fixed concentration (10 ppm) the current response level of the sensor has not changed significantly. Average response i.e. (sensitivity for a fixed ppm) of the sensors for 10 ppm ammonia concentration is found to be 86.8 ± 1.1 (%) for 10 cycles. The current response data for a typical sensor of 10 cycles is shown in Fig. 6. Beyond initial few cycles, the response stabilizes to within 10% after repeated cycling.

### Response time

**Figure 7:**
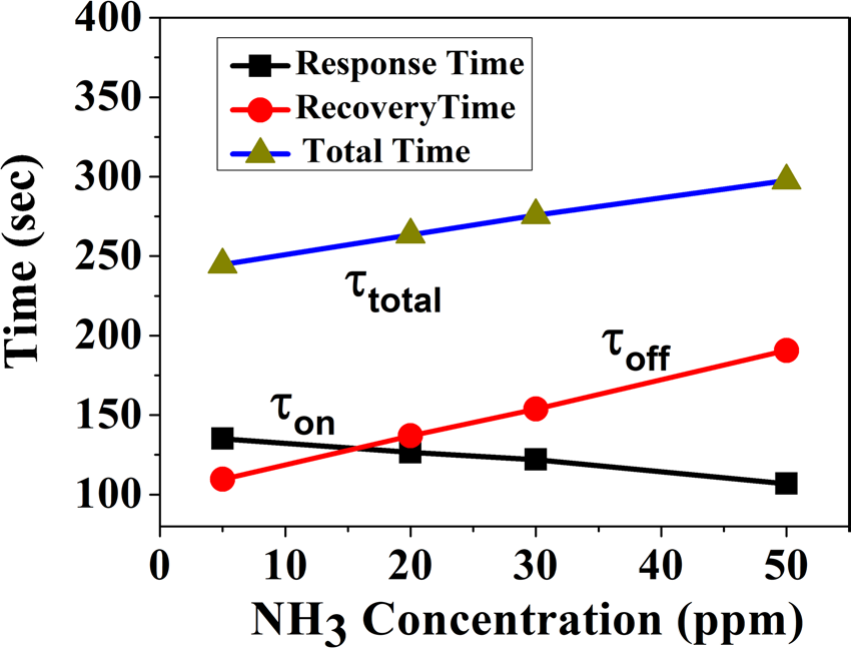
Response time τ_ON_ (ON cycle) and recovery time τ_OFF_ (OFF cycle) of the sensor or as a function of NH_3_ gas concentration.

One of the important parameter in the sensor operation is the time of the sensor to respond (response time) when the gas is turned ON which we refer as *τ*_*ON*_. This is the time the sensor takes to reach 90% of the maximum final response output. We have also measured the time for the sensor to recover after the gas is turned OFF. This we refer to as recovery time (*τ*_*OFF*_). This is the time at which the sensor resistance takes to recover to within 10% of the initial value. In Fig. 7 we show the *τ*_*ON*_ and *τ*_*OFF*_ as function of the NH_3_ concentration for a typical sensor. (The values of *τ*_*ON*_ and *τ*_*OFF*_ are reproducible from cycle to cycle and within ±10% of the average value.) An important parameter is *τ*_*tot*_ ≡ *τ*_*ON*_ + *τ*_*OFF*_ which is the minimum total time the sensor takes to respond and recover, when the gas is turned ON and OFF. This is also plotted in Fig. 7.

Figure 7 shows that the response time of the sensor decreases with increase of concentration of NH_3_ gas, whereas, the recovery time increases with increase of NH_3_ gas concentration. A likely reason for shorter response time for higher concentration is due to availability of larger number of NH_3_ gas molecules that get adsorbed on the sensor surface reacting with the MAPI film. Similarly the longer recovery time for higher concentration can be attributed to larger areas of reacted surface at higher ppm level that need to be recovered on removal of the gas. Interestingly *τ*_*tot*_ is lowest at 10 ppm (~250 sec), increases steadily to 300 sec for 50 ppm. This primarily occurs because the recovery time increases rather rapidly for higher concentration. The response time does decrease on heating during recovery. However, we would like to avoid any heating to our sensor so that the sensor can act as an unheated room temperature sensor. In particular, since the response is lowest for low concentration, the sensor is optimized for operation with low concentration of NH_3_ gas.

### Noise limited detection: sensitivity below 1 ppm

For a good sensor, limit of detection is an important issue. We have tested the sensor for exposure to NH_3_ concentration of 1 ppm. (Note: We don’t test the sensor directly below 1 ppm due to lack of calibration facility below this level). The sensor, however shows response well below 1 ppm. Data are shown in Fig. 4(c). The change in current of a given device is ≈0.15 nA for 1 ppm gas concentration. From the observed current sensitivity at 1 ppm and the current noise level (rms current noise) we can estimate a noise limited detection limit of the sensor.

**Figure 8:**
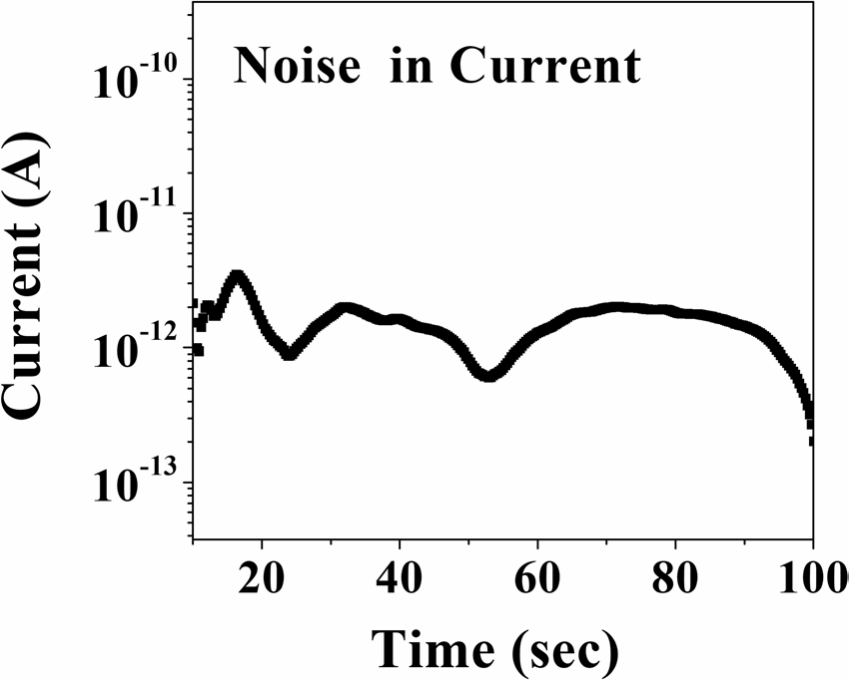
The current noise of the sensor.

The measured rms noise in the current for the given device is ±1.6 pA The noise in current is shown in Fig. 8. Thus the current noise limited detectability of the sensor is around ±10 ppb. Though the calibrated sensitivity is down to 1 ppm, the sensor has sensitivity much below 1 ppm as observed from the data limited by current noise. Such level of sensitivity would make the sensor suitable for diagnostics using exhaled breath analysis where a sensitivity of 100 ppb or better is needed.

### Effect of humidity on sensing property

**Figure 9:**
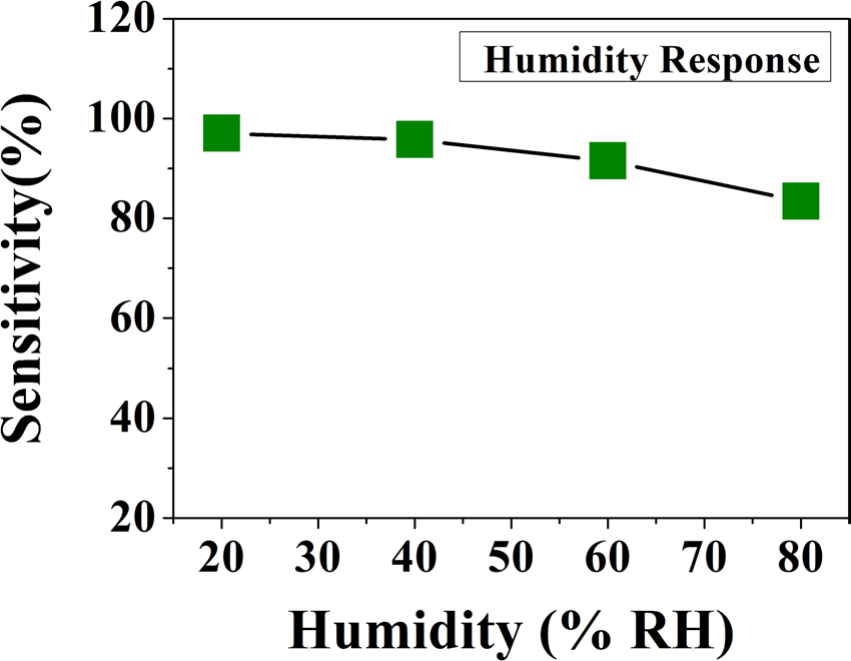
Dependence of the response of the senor on humidity (Test carried out for fixed concentration of 20 ppm of NH_3_ gas).

The effect of humidity on the sensor was studied by injecting water vapor in the test chamber by keeping the other parameters constant. The amount of water vapor was measured by using a humidity meter. This allowed the relative humidity (% RH) of the test chamber to be controlled effectively. A fixed NH_3_ concentration (20 ppm) was then admitted. Figure 9 shows the response of the sensor to 20 ppm NH_3_ in different relative humidity ranging from 20% RH to 80% RH.

It has been observed that sensitivity decreases, although by a small measure, with increase of humidity in this range. The change of sensor response in the range of observed humidity is nearly 15%. An uncertainty in response of about 15% would imply an uncertainty of about 10 ppm in NH_3_ concentration. If an uncertainty of 10 ppm is acceptable no humidity control is needed. For precise NH_3_ concentration determination as needed for breath analysis with an uncertainty of better than 1 ppm a humidity control in the test chamber to better than 20% of RH will be necessary.

### Selectivity of the sensor towards NH3 gas

**Figure 10:**
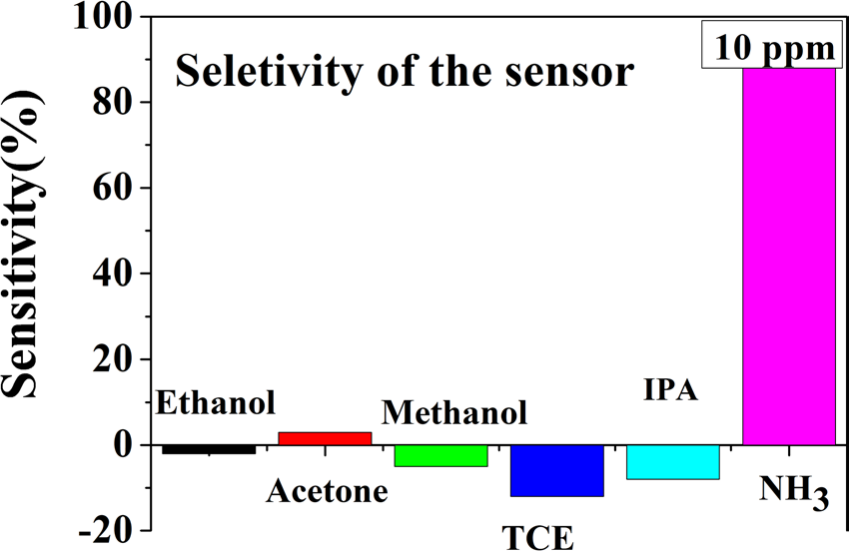
Selectivity of the sensor to NH_3_ gas (fixed concentration of 10 ppm) and other organic volatiles (saturated vapours).

For practical use selectivity is an important parameter for any gas sensor. The sensor was tested for different pure volatile organic vapours that include ethanol, methanol, acetone, 2 propanol (IPA), Trichloro ethelyne (TCE) vapors, etc. Results are given in Fig. 10. The test was done with saturated vapor of the organic volatiles. It is noted that the sensitivity towards NH_3_ is much higher as compared to other species of test gases. More importantly in case of NH_3_ the sensor resistance decreases on exposure to NH_3_. For most gases tested (barring Acetone) the sensor resistance increases on exposure to the gas leading to negative *S* (according to Eq. 1). Thus the change occurs for other gases are qualitatively different leading to a good selectivity for NH_3_ gas.

### Stability of the sensor paper towards storage

**Figure 11:**
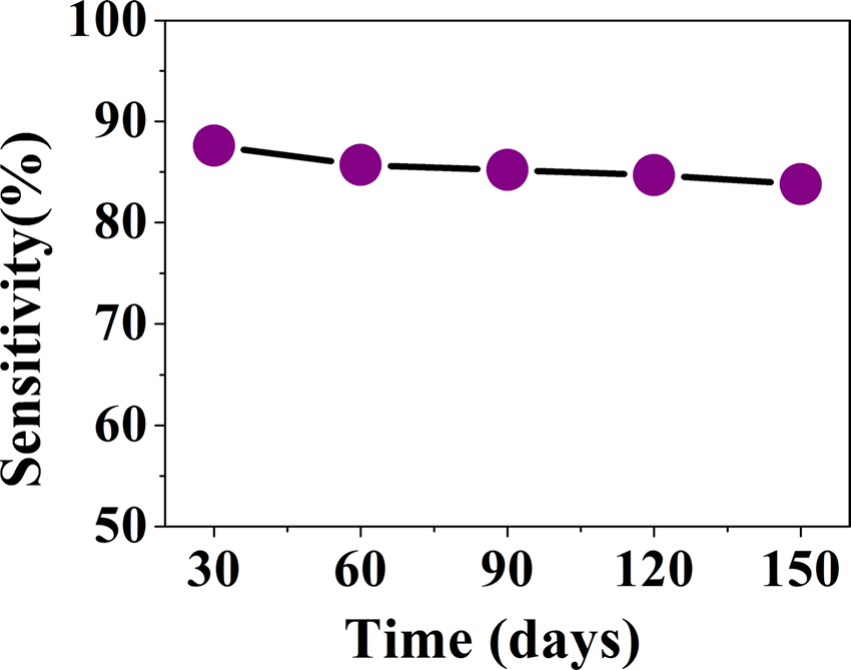
Stability study of the sensor for a fixed concentration of 10 ppm NH_3_ gas.

A collection of sensor papers were stored in a pumped desiccator. At an interval of 30 days one strip was taken out and then exposed to only a fixed concentration (10 ppm) of NH_3_ gas in the test chamber and sensitivity of the sensor was measured at room temperature. This was continued for a period of 150 days. The data are shown in Fig. 11.The decrease of the sensor response at 10 ppm NH_3_ concentration for storage over a period of 150 days is <5%. After some initial degradation over 3 weeks the sensor response stabilizes within 2–3%. A good shelf -life and almost constant sensing performance of the sensor shows that the paper electronics based MAPI sensor qualifies as a stable sensor for NH_3_ gas at room temperature.

### Effect of bending on sensing property

**Figure 12:**
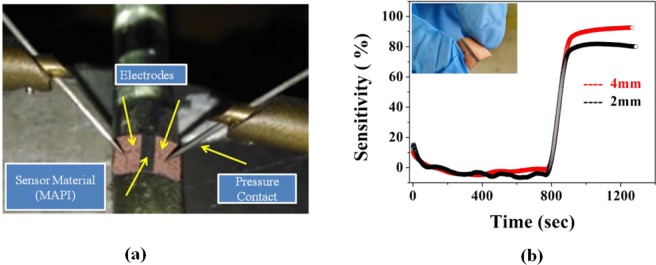
(**a**) Photograph of the flexible sensor at bend condition (**b**) Performance of the sensor at different bend conditions.

Mechanical flexibility is important for portable substrates. To study the flexibility of the paper sensor; we have checked the sensitivity at different bending condition varying the bending radius. The data for two different bending radii for fixed NH_3_ concentration is given in Fig. 12. From Fig. 12 it is observed that the sensor is capable to detect NH_3_ gas at bend condition also. Sensing performance is not much affected due to bending. At higher bending radius sensitivity is slightly decreased as effective exposed area to ammonia gas has been reduced. A change ~10% has been observed due to highest bending (bending radius ~4 mm).

## Discussions

### Comparison with other existing NH3 sensors

The sensor reported here is an unheated sensor. All sensing measurements were carried out at room temperature (27 °C). Other usually known NH_3_ sensors, such as metal oxide based sensors need elevated temperature [200°–500 °C] for operation with adequate sensitivity^2^. The heated operation immediately raises the operational power requirements. In heated NH_3_ gas sensors based on metal oxide as active materials the typical sensitivity ~25% at NH_3_ concentration 1000 ppm at operation temperature 200 °C^10^. PANI based flexible NH_3_ sensors are reported that work at room temperature. But these sensors have significantly less sensitivity (e.g, sensitivity of 30% at 200 ppm). In comparison to other sensors the one reported here based on paper and using MAPI as active material have much higher sensitivity, selectivity and relatively fast response considering the fact that it operates at room temperature. Since no heating is needed for its operation, its operational power requirement is only few nanoWatt making it compatible with most portable electronics that are also cloud compatible. The sensor is also cheap to fabricate being paper based and due to utilization of a cost effective simple wet route chemistry based fabrication process.

### Sensing mechanism

The mechanism of gas sensing in solid state gas sensors (like the one based on metal oxides) depend on redox mechanism^1^. This mechanism is not applicable in sensors based on MAPI as the sensing material. It has been established by us in a previous publication^12^ in context of visual NH_3_ sensing using MAPI as the sensing material, that exposure to NH_3_ gas leads to decomposition of MAPI to PbI_2_ that can be detected by its appearance of characteristics color. A number of characterization tools were used to establish this. The visual color change is detectable when the exposure is above 10 ppm. When the exposure is for a short time the process is reversible if the exposure is below 50 ppm. At higher concentration of NH_3_ and for longer exposure the decomposition becomes irreversible.The mechanism of detection that there is decomposition of MAPI on exposure to NH_3_ gas thus makes the process strongly specific to NH_3_ gas detection.

The electrical detection process is more sensitive. Exposure to even a small amount of NH_3_ leads to detectable change in the device current. This signifies that the conductivity of the thin film of the active materials increases on gas exposure.

**Figure 13:**
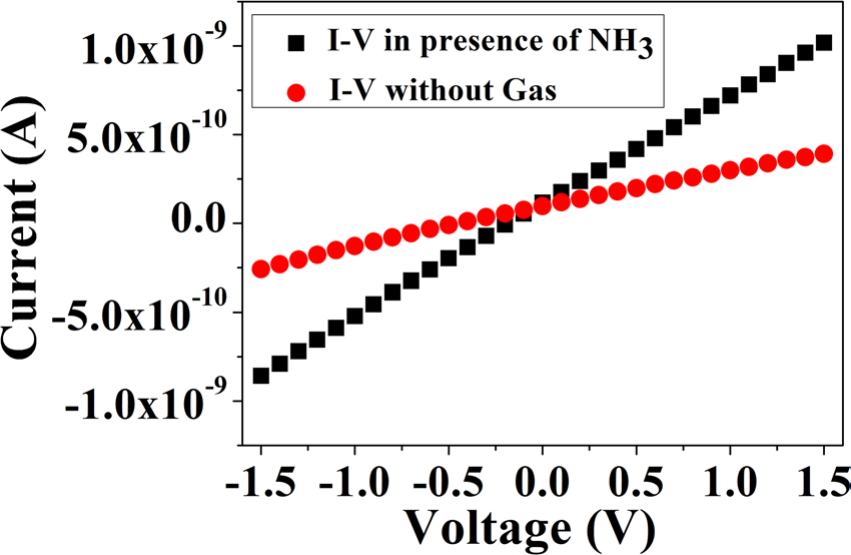
Comparison of I-V data of the unexposed sensor and the exposed sensor in NH_3_ gas (~10 ppm).

We have checked this also from the I-V characteristics of the device in presence of NH_3_ gas. Comparison of I-V data of unexposed sensor and exposed sensor in NH_3_ gas (for 10 ppm concentration) is shown in Fig. 13. The data shows the nature of current-voltage relationship remains same in presence of NH_3_ gas. The conductivity is increased on gas exposure leading to slope change in the I-V Characteristics. This happens due to appearance of PbI_2_ on exposure to NH_3._

As stated before in our earlier work^12^ on visual sensor we established that on exposure to NH_3_ there is partial conversion of MAPI to PbI_2_ which has higher conductivity than MAPI. For concentration ≤10 ppm, the change is reversible as established in the visual sensor work. Observation of saturation of sensitivity on exposure to higher concentration of NH_3_ would arise when a larger volume fraction is exposed and decomposed. At low concentration the phenomena may be surface dominated. Since surface diffusion is faster, one would expect reasonable response time even in unheated operation.

## Conclusions

In summary, we have demonstrated a cheap and paper based highly sensitive NH_3_ gas sensor that operates at room temperature. The sensor is based on a new sensor material perovskite halide MAPI. The sensor has a sensitivity of 55% even at exposure to only 1 ppm of NH_3_ and the current noise limited detectibility is ~10 ppb. The sensor also has very high selectivity. This makes the sensor very useful for application in exhaled breathe analysis for disease diagnosis in addition to more conventional use in sensitive monitoring of work places for hazardous gas leaks.

## Experimental Section

### Growth of the MAPI on paper

The sensor was fabricated by following simple wet chemistry route. Synthesis of MAPI was done by standard method by mixing hydro iodic (HI) acid with ice cooled methyl ammonium (CH_3_NH_2_) solution to form methyl ammonium iodide (CH_3_NH_3_I/MAI). Details are given in Supplementary Notes. The initial step was to make a saturated solution of lead iodide (PbI_2_) in dimethylformamide (DMF). The solution was then dip coated on a commonly used paper for ~30 sec. After oven drying, the dip coated paper was immersed for 24 hrs in a solution of CH_3_NH_3_I in Iso- Propyl Alcohol (IPA). This leads to formation of film of MAPI microrods on the paper. The black colored methyl ammonium lead iodide (MAPI) coated paper is dried and it is ready to use. The process is fast, energy efficient and is compatible with large scale production.

### Device fabrication

**Figure 14:**
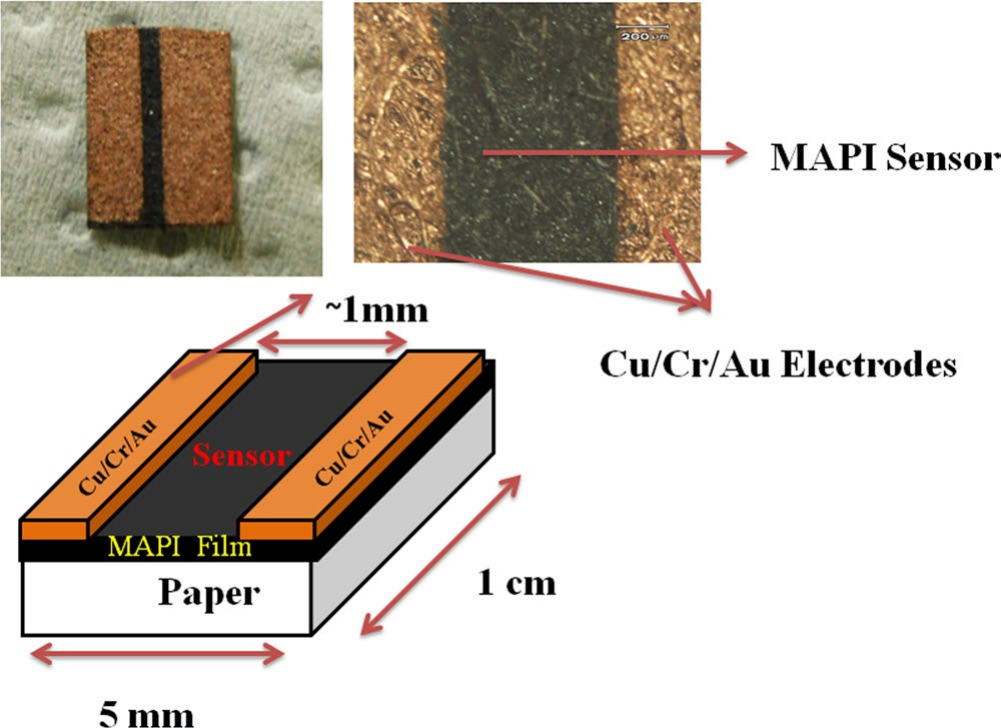
Schematic of the Sensor device with photograph of the actual device.

As prepared MAPI coated paper was cut into small pieces (typical size of 1 cm × 5 mm) to form the device by evaporation of two electrodes. The typical channel length is ~1 mm. Schematic of the sensor is shown in Fig. 14. Metallization was done by thermal evaporation of Cu and Cr/Au pads in sequence through a metal mask to make the electrodes. Cu was chosen as electrodes to make the contact ohmic. The top Cr/Au layers protect the Cu electrode from oxidation.

### System for testing the sensor and calibration of gas concentration

A custom made set- up was made to characterize the sensing properties and the sensor was placed in test chamber. The test chamber can be pumped down to a pressure of 10^−6^ mbar by a turbo pump. During the experiment, controlled amount of NH_3_ gas was mixed with known volume dry Nitrogen (N_2_) gas in the chamber, which allows controlled testing for very low concentration of NH_3_ gas. *I*–*V* and *I*–*t* measurements were done using a Source-Meter employing a two probe configuration and custom-developed computer programs. All sensing measurements were performed at room temperature (27 °C). Spring loaded clips were used for making contact to the electrodes of the paper based sensor.

## Supplementary information

Supplementary Information
