# Supplementary material for: High sensitivity NH3 gas sensor with electrical readout made on paper with perovskite halide as sensor material
Source: Sci Rep. 2019 May 23;9:7777. doi: 10.1038/s41598-019-43961-6 (PMC6533271; doi:10.1038/s41598-019-43961-6)
Supplement: Supplementary file 1 — Supplementary Information [file 41598_2019_43961_MOESM1_ESM.docx]

Supplementary Section of the manuscript entitled as

**High sensitivity NH_3_ gas sensor with electrical readout made on paper with perovskite halide as sensor material**

Avisek Maity^1^, A.K. Raychaudhuri^1, 2^, Barnali Ghosh^1, 2*^

^†^ Department of Condensed Matter Physics and Materials Sciences, S.N. Bose National Centre for Basic Sciences, JD Block, Sec-III, Salt Lake, Kolkata-700106, India

^‡^Technical Research Centre (TRC), S.N. Bose National Centre for Basic Sciences, JD Block, Sec-III, Salt Lake, Kolkata-700106, India

* Corresponding Author

Email: barnali@bose.res.in

**Synthesis of MAI (CH_3_NH_3_I)**

MAI synthesis consists of three major steps:

Synthesis of methyl ammonium iodide provided by the reaction of hydro Iodic (HI) acid with ice cooled methyl ammonium solution.

Initially, HI acid added slowly to ice cooled methyl ammonium solution under stirring for 3-4 hrs until a dark brown solution of methyl ammonium iodide has been formed.

CH_3_NH_2_+HI CH_3_NH_3_I

In the next step the dark brown colored solution was heated for an hour at 90°C and cooled keeping at room temperature. Then cold diethyl ether was added to clean it. A color less crystal forms. The cleaning process using diethyl ether is done repeatedly to achieve the colorless crystal completely from the solution.

In the final step, the colorless crystals are redissolved in Ethanol and cold ether is added until the whole colorless solution transformed to white precipitation powder. Then the white powder was taken out from filter paper for vacuum annealing (at 70°c for 24 hrs). This was the final product of CH_3_NH_3_I.

Supplementary Fig: 1(a)

XRD of Paper substrate





Supplementary Fig 1(a): XRD of paper substrate

Supplementary Fig: 1(b)



XRD of MAPI film

Supplementary Fig 1(b): XRD of MAPI film

Supplementary Fig: 2(a)

XRD of MAPI film before ammonia exposure:





Supplementary Fig 2(a): XRD of MAPI film before ammonia exposure

Supplementary Fig: 2(b)

XRD of MAPI film after removal from ammonia exposure:





Supplementary Fig 2(b): XRD of MAPI film after removal from ammonia exposure

Supplementary Fig 3:

Schematic of test Chamber
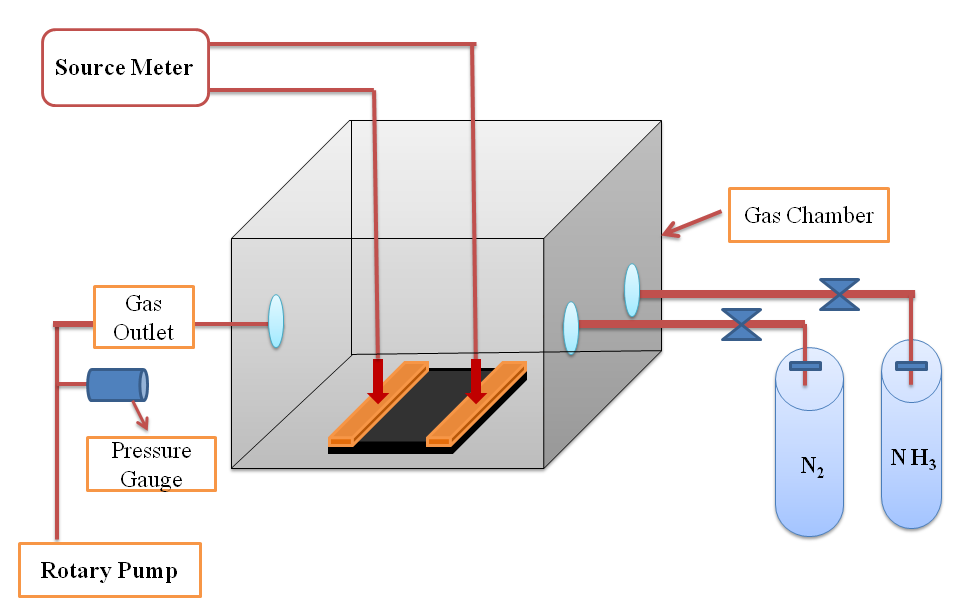


Supplementary Fig 3: Schematic of the test chamber
